# Silent Victories: The History and Practice of Public Health in Twentieth-Century America

**DOI:** 10.3201/eid1312.071159

**Published:** 2007-12

**Authors:** Stephen S. Morse

**Affiliations:** * Columbia University, New York, New York, USA

**Keywords:** public health, history, medicine, history, public health, practice, book review

The 20th century witnessed some notable public health triumphs in America: improvements in the water supply, further control of several infectious diseases through vaccines and antimicrobial drugs, and increases in life expectancy with enormous improvements in survival rates of mothers and their infants. What made these improvements possible? For anyone who has ever wondered, this book is an excellent place to start looking for answers.

The stated purpose of the book is not to provide a comprehensive history of public health in America but to discuss 10 key public health advances of the 20th century. This is a broad objective in itself, which this volume richly achieves. The advances, originally chosen for MMWR (Morbidity and Mortality Weekly Report) in 1999, are each expanded into a section of the book: Control of Infectious Diseases, Control of Disease through Vaccination, Maternal and Infant Health, Nutrition, Occupational Health, Family Planning, Fluoridation, Vehicular Safety, Cardiovascular Disease, and Tobacco and Disease Prevention.

The facts and figures are all there, of course, and they are generally very well presented and referenced. Infectious diseases are well represented; their respective chapters are excellent and informative. But it would be a pity if the reader stopped there. A unique strength of the book is the pairing of these expository chapters with essays by social scientists and historians who explore aspects of the social or political context. This combination makes it a book to savor. Experienced practitioners having a hard day may be encouraged to learn that many public health triumphs we take for granted today (the apt title Silent Victories is from a 1923 lecture by C.-E.A. Winslow) were made possible only by heroic and sustained effort.

One theme that emerges is the importance of coalitions, often including not only the medical community and health departments (and sometimes industry), but also activists, reformers, and even ordinary citizens who became passionate about a cause. Getting recognition and consensus within the medical community was essential, and not always easy, as in the development of occupational health, or even pasteurization at first. Wolf’s article, for example, notes that ensuring clean pasteurized milk required 30 years of effort, during which time many infants died. In traffic safety, discussed by Albert, the activists were often the ones who pushed government into taking action. With regard to the more recent efforts toward tobacco cessation, Brandt argues that the 1964 Surgeon General’s Report was a watershed comparable to John Snow’s work on cholera, as it developed the foundations not only for tobacco cessation but also for chronic disease epidemiology.

But, of course, public health cannot rest on these laurels. As Koplan and Thacker note in the Epilogue, public health in the coming century will face many challenges. Some are a continuation of 20th-century trends, such as emerging infectious diseases, healthy lifestyle choices, and ensuring that basic public health measures are available globally. Others will be new, including the aging of large segments of the population. As this book demonstrates, one of the best ways to meet the new challenges may well be to fully appreciate how these past successes were achieved.

